# Immune Prophylaxis and Therapy for Human Cytomegalovirus Infection

**DOI:** 10.3390/ijms22168728

**Published:** 2021-08-13

**Authors:** Evi B. Struble, Haruhiko Murata, Takashi Komatsu, Dorothy Scott

**Affiliations:** 1 Division of Plasma Protein Therapeutics, Office of Tissues and Advanced Therapies, 10903 New Hampshire Avenue, Silver Spring, MD 20993, USA; Dorothy.Scott@fda.hhs.gov; 2 Division of Viral Products, Office of Vaccines Research and Review, Center for Biologics Evaluation and Research, 10903 New Hampshire Avenue, Silver Spring, MD 20993, USA; Haruhiko.Murata@fda.hhs.gov; 3 Division of Antivirals, Office for New Drugs, Center for Drugs Evaluation and Research, United States Food and Drug Administration, 10903 New Hampshire Avenue, Silver Spring, MD 20993, USA; Takashi.Komatsu@fda.hhs.gov

**Keywords:** cytomegalovirus therapy, cytomegalovirus prophylaxis, animal models for cytomegalovirus

## Abstract

Human Cytomegalovirus (HCMV) infection is widespread and can result in severe sequelae in susceptible populations. Primary HCMV infection of naïve individuals results in life-long latency characterized by frequent and sporadic reactivations. HCMV infection elicits a robust antibody response, including neutralizing antibodies that can block the infection of susceptible cells in vitro and in vivo. Thus, antibody products and vaccines hold great promise for the prevention and treatment of HCMV, but to date, most attempts to demonstrate their safety and efficacy in clinical trials have been unsuccessful. In this review we summarize publicly available data on these products and highlight new developments and approaches that could assist in successful translation of HCMV immunotherapies.

## 1. Introduction

Human CMV (HCMV) or human herpesvirus 5, is the largest of the human herpesviruses, with double stranded linear DNA of up to 240 kbp encoding for at least 166 proteins [1]. It is ubiquitous, with an estimated one in three US children encountering it by age five, and more than 50% of the adult population having done so by age 40 [2]. An even higher prevalence has been shown in South America, Africa and Asia [3]. Modes of transmission include direct person-to-person contact, maternal-to-child transmission (ante-, peri- and postnatally), and through organ and cell transplantation. Although usually subclinical in immunocompetent hosts, HCMV infection is often accompanied by prolonged, even life-long viral shedding through saliva, urine and other bodily fluids [4]. In susceptible individuals, including the immune suppressed (such as transplant recipients [5]), and infants that acquired the virus prenatally [6], the infection can result in severe sequelae. There is no vaccine for HCMV, and, although effective, small molecule antivirals are often associated with resistance and breakthrough infection. Thus, vaccines and biologics, such as polyclonal and monoclonal antibodies, hold great promise in HCMV prophylaxis and therapeutics.

## 2. Virology

HCMV can infect a diverse number of cells resulting in productive viral replication in epithelial, endothelial, fibroblasts, hepatocytes, muscle and neuronal cells [7]. Following primary infection, HCMV finds its way to the hematopoietic progenitor cells in the bone marrow where it enters a life-long latency. Sporadic reactivation from latency is believed to be frequent and can occur in response to physiologic processes including lactation [8] or inflammatory signaling [9]. The mechanistic underpinnings that underlie latency and flare-ups are under active investigation, with miRNA regulators thought to be playing a role [9].

HCMV initially tethers to host-cell surfaces through the interactions of viral envelope glycoprotein (g) gM/gN complexes with cell heparan sulfate proteoglycans [10]. Following this interaction, the virus transitions to a more stable binding via gB [11,12]. In a post-attachment step in the entry pathway, gB interacts with cellular integrins to trigger virus-cell fusion in all tested cell types [13,14,15]. In concert with gB-receptor interactions is the engagement of gH/gL containing complexes with cognate receptors. In contrast to gB-host cell interactions, which are required for entry into all physiologically relevant cell types, gH/gL can form two distinct complexes capable of mediating entry into distinct cell populations. The gH/gL/gO complex is essential to mediate entry into fibroblast cells, whereas the gH/gL complex comprised of gH/gL/UL128/UL130/UL131 (termed the pentameric complex) is essential to mediate entry into myeloid, epithelial, and endothelial cells [16,17]. In addition to playing essential roles in mediating viral entry into host cells, the gB and pentameric complex (PC) are both required to mediate cell−cell fusion events important for the transfer of virus between monocytes and endothelial cells and the dissemination of virus [16,18,19].

Both gB and gH/gL are conserved across the Herpesviridae family [20,21]. gB binds to various integrins via the disintegrin-like domain, or to epidermal growth factor receptors to promote entry, and likewise, gH/gL can bind to integrins [12,14,20,22]. Both gB and gH/gL are required to enter into all cell types, and neither complex alone is sufficient for entry. gH and gL associate with gO to facilitate HCMV entry into fibroblasts, whereas PC mediates entry into all other cell types [20,23]. However, PC is dispensable for entry into fibroblasts [24]. These surface glycoproteins are an important target for antibodies and are potential vaccine antigens. Following natural infection, the majority of neutralizing antibodies that inhibit infection of epithelial/endothelial cells target PC [25].

Laboratory strains of HCMV that have been extensively passaged on fibroblasts lose their ability to infect epithelial and endothelial cells [17]. This loss of tropism has been mapped to the UL128–131 locus [17]. Additionally, a great degree of variability can be found in these and naturally occurring, widely circulating strains. Multiple genotypes, as well as “mixing and matching” of glycoproteins of different genotypes in a single recombinant virus have been shown and were recently reviewed [26]. Such polymorphism, especially when it occurs in surface glycoproteins and their complexes, gives rise to strain-specific immune responses that often do not provide protection from re-infection from a mismatched strain. Thus, the selection of the specific sequence(s) to use in a vaccine, or the geographical location and size of the donor pool for manufacturing hyperimmune globulin products become critical considerations in the search for effective prevention and therapies for HCMV.

### Animal Models

Animal models of disease are an essential tool for in depth studies of viral diseases, as well as for testing vaccine and treatment approaches prior to commencing clinical trials. Administering experimental pharmaceuticals in such models would, for example, confirm or reject hypotheses underlying their pharmacologic activities and help derive a dose expected to be beneficial in the intended patient population. Unlike other common human viruses (including influenza virus and coronaviruses) which can infect select mammalian species, no such permissive hosts have been identified for HCMV. Species-specific CMVs have been described and characterized for a number of commonly used laboratory species, including non-human primates (NHP), guinea pigs, mice and rats. These viruses have a strict species selectivity. Accidental infections notwithstanding, there is no experimental evidence of human or other mammalian CMVs (including those from closely related NHP species), crossing the species barrier [27]. In the absence of an animal infection model, different approaches have been used to understand the mechanisms of HCMV infection and re-infection as well as to identify potentially efficacious therapies.

Rhesus monkey (*Macaca mulatta*) CMV (RhCMV) infection recapitulates many of the clinical and genetic hallmarks of HCMV [28]. In addition to a similar natural history of infection in their respective hosts, the two viral species share a high degree of similarity in genome length and structure and contain a large proportion of orthologous ORFs [29]. Proteins that mediate viral entry into susceptible cells and are immunodominant for neutralizing antibody responses, such as gB and the pentameric complex (PC), display some sequence conservation and, perhaps more importantly, mediate similar cellular entry and tissue tropism pathways [30]. Immunizing rhesus monkeys with vaccine candidates, such as those that express RhCMV gB (among other subunits) [31] and PC [32], resulted in neutralizing antibody response and a reduction in serum viremia following challenge. Thus, these studies provided proof-of-concept data that similar approaches may confer benefit in clinical trials.

A similar species-specific approach has been used to assess the pharmacologic activity of human polyclonal hyperimmune globulin (HIG) products. For example, serum from rhesus macaques seropositive for RhCMV [33] was used to make HIG preparations analogous to HCMV HIG, which are made from human plasma and used for the prevention of CMV disease associated with solid-organ transplantation. The RhCMV preparations were then used to evaluate if they could prevent vertical transmission of primary infection when administered to an NHP model of congenital CMV (cCMV), specifically CD4-depleted pregnant macaques infected with RhCMV. The HIG was administered at doses equal to or higher than those used in clinical trials with the human products. While all (6/6) dams in the untreated control group vertically transmitted RhCMV infection, only 2/3 in the “standard” and 0/3 in the “high potency” RhCMV HIG groups did so, indicating that HIG preparations have the potential to reduce the rate of cCMV infections [33]. Al-though the differences in HIG dosing amplitude and frequency precluded head-to-head comparisons between the “standard” and “high potency” preparations, these data suggested that a more potent HIG preparation, when given at a higher and/or repeated dose, has the potential to reach 100% efficacy.

A widely used animal model of CMV disease and infection is the guinea pig (GP). GPCMV is phylogenetically more distant from the human virus compared with RhCMV. Nevertheless, there are parallels between human and guinea pig viruses, including widespread lifelong infection in commercially available guinea pig colonies, development of severe infection in the presence of immune suppression, as well as vertical transmission of primary infection during pregnancy with associated sequalae [34,35]. Furthermore, it has been proposed that, due to a high degree of conservation in the immunodominant region of gB, the guinea-pig model can be used to assess vaccine or antibody therapies that target this specific region of the protein [29]. It should be noted that antibodies against the conserved region of gB represent only a fraction of anti-CMV immune repertoire following natural infection. Thus, even if useful for a subset of prophylactic and therapeutic modalities, the model may not account for the entire spectrum of human antibody responses to CMV infection and the associated protective mechanisms.

Mouse CMV (MCMV) is another model virus that has been widely used to investigate HCMV infection and disease. The utility and limitations of this model have been recently reviewed [36], so we will not describe this model in detail. We will, however, draw attention to recent developments with humanized mouse models that contain a functional cellular and humoral human immune system (HIS) [37]. These models represent a significant advantage and lay the groundwork for animal studies that use HCMV and not the murine homolog. Several versions of HIS humanized mice have been developed and are available commercially. They use strains of mice with multiple severe deficits in their autologous immune system, such as NOD/scid/gamma mouse (NSG Jackson Labs) and equivalent NOG and NRG mice (Jackson Labs and Taconic). These strains are then transplanted with human myeloid cells, often following irradiation or pharmacologic treatment to further ablate the endogenous immune system and ensure the engraftment of a functional HIS [37]. An example of successful application of this approach is bone marrow/liver/thymus (BLT) humanized mice surgically transplanted with human fetal liver and thymus tissue and infused with CD34+ cells, which engraft in the bone marrow.

**Figure 1:**
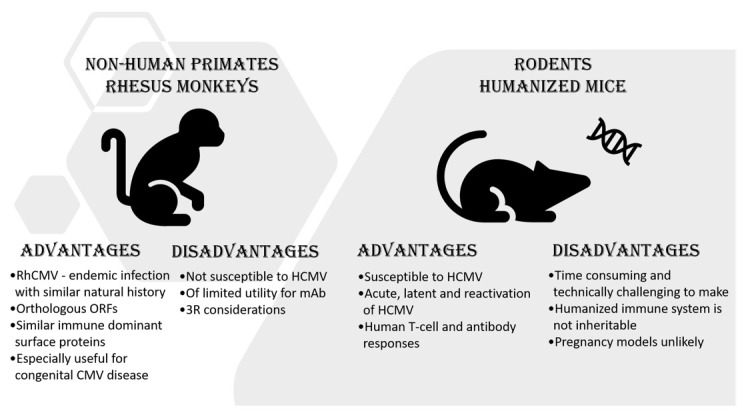
Animal models for CMV infection and disease.

In recent decades, HIS models have been used to recapitulate latent and chronic infection, study immune mechanisms, and assess therapies directed at viruses, including HIV [38] and HCV [39]. They are now successfully being tested with HCMV [40,41]. Working with such models, researchers have captured HCMV acute infection, the development of virus-specific T-cell and antibody responses and the establishment of latency and reactivation. Although promising, the limitations of HIS mouse models (some of which are shown in Figure 1) have precluded their wide adaptation in HCMV research. For example, generating these models is labor intensive and requires the application of advanced surgical skills. In addition, not all elements of HIS are equally expressed, with monocytes and macrophages lagging to a large extent [42]. Given the role that cells of macrophage lineage play in latency and, especially, reactivation of HCMV, this limitation can contribute to the reduction in viral load and spread seen in the humanized mouse compared to the human host [41].

As summarized in Figure 1, commonly used and emerging animal models have advantages and disadvantages when used to study HCMV infection and disease. Further research into these models should facilitate and promote the search for safe and efficacious vaccines and treatments. 

## 3. Immune Prophylaxis and Therapy

For the purpose of this review, antiviral immunotherapy is the use of vaccines or antibody preparations to treat or, in the case of immune prophylaxis, prevent viral disease. We will also discuss the role of the vaccines in stimulating protective cell-mediated immune responses, an important component of effective vaccines. However, virus-specific T-cell immunotherapy, recently being tested and showing promise in the prevention and treatment of infections, including CMV in a transplant setting [43,44], is beyond the scope of this paper and will not be discussed. (CMV and HCMV will be used interchangeably for the rest of this review.)

The best vaccines stimulate a broad immune response that includes both antibodies and immune cells. The goal is to elicit a response that is effective and long lasting, given the temporal lag between vaccination and exposure to the pathogen. This can only be achieved if memory B and T cells, which can quickly reactivate and expand to combat infection, are made. The role of immune cells in long-lasting and effective protection notwithstanding, the ability of vaccines to elicit high levels of neutralizing antibodies is often one of the most important correlates of protection.

Antibodies, or immunoglobulins (Ig), are produced and secreted by plasma cells, the terminally differentiated B cells residing in peripheral lymphoid tissues. Antibodies are multi-functional molecules, combining the ability to bind to an antigen (in this case the virus or its components) through the complementarity-determining regions (CDRs) in the variable domain of the antibody with effector functions through their constant (Fc) domain. The effector functions of the antibody ultimately mediate the control or clearance of the viral infection and have been proposed also to play a role in modulating immune response [45].

The antibody molecules most effective in immune prophylaxis and therapy have undergone class-switching from class (isotype) M antibodies, characteristic of an immature immune response, to class G (IgG) antibodies. The best therapeutic IgG molecules have also undergone affinity maturation, a process by which naïve B cells, through somatic hypermutation, have acquired the ability to produce antibodies with high affinity for the viral antigen [46,47]. As crystal structures of antibody-antigen complexes combined with large-scale sequencing data have shown, this high affinity is achieved through several discrete strategies that include better shape fitting and improved energetic and entropic components of the interaction between the CDRs and the viral antigen [48].

While vaccination is, by nature, polyclonal, antibody products for immunotherapy can be polyclonal or monoclonal. Polyclonal preparations contain a mixture of IgG molecules isolated from a pool of plasma donors or from animal plasma. The sequence of each individual molecule can be different in their constant and variable regions, as these preparations often contain all four subclasses of the IgG found in human plasma. As a result, these products can both bind a diverse set of viral epitopes and invoke multiple effector pathways, depending on their subclass. In contrast, each monoclonal product contains a single kind of pathogen-specific antibody made from the culturing of mammalian cells at industrial scale. Differences in the post-translational modifications and other production-related factors notwithstanding [49], all the molecules in such preparations have the same sequence, structure and function.

Although neutralizing antibodies are believed to be the most important correlate of protection for most vaccines, recent analyses of samples from non-clinical studies [50] and clinical trials [51,52] with HCMV gB vaccine have uncovered an important role of non-neutralizing antibodies in protection from infection. Although no antibody-dependent cellular cytotoxicity (ADCC) activity was detected in vaccinated subjects [51], it was hypothesized that the mechanisms of protection following viral challenge may be related to antibody-dependent cellular phagocytosis (ADCP) or ADCC by macrophages and NK cells, respectively. Studies, such as those performed in animal models challenged with HCMV, should provide more insights into the mechanisms underlying these observations.

We interrogated the published literature and clinicaltrials.gov for antibody-based immunotherapies and vaccines under development for CMV prevention and therapy. Although an area of continued development, especially in the preclinical stage [53], there are no vaccines or monoclonal antibody therapies approved for the treatment of CMV infection. As shown in Table 1, only one polyclonal preparation, a human plasma derived product, Cytogam [54], has been approved for use in the US. As many as twelve active clinical trials of vaccine candidates are registered in clinicaltrials.gov, and other vaccines and antibody therapies likely are still in research and discovery stages. The following sections will discuss these prophylactic and treatment modalities, including upcoming developments, in more detail.

### 3.1. Vaccines

Despite efforts spanning decades, with a total of 21 clinical trials that are ongoing or were completed in the last ten years (according to clinicaltrials.gov, Table 1), a licensed CMV vaccine is not yet in existence. An influential cost-benefit analysis in 2000 by the National Academy of Medicine ranked CMV to be of the highest tier priority as a target for vaccine development, largely based on the public health burden caused by congenital CMV infection [55]. The CMV disease burden among immunocompromised individuals (for example, transplant patients) calls attention to another group for whom vaccination may be valuable. Assuming the broad availability of a safe and effective vaccine, routine vaccination in younger individuals and children can also be envisioned for the purpose of reducing CMV transmission and the exposure of susceptible individuals, such as pregnant women. The species-specific nature of CMV and the lack of an animal model that recapitulates all relevant aspects of CMV pathology in humans have hampered vaccine development. Nevertheless, in vivo models particularly using guinea pig CMV and rhesus macaque CMV, have yielded valuable mechanistic insights [29]. Immune correlates of protection have not been identified and likely vary depending on the target population and indication for vaccination, although both humoral and cellular immune responses are likely to play important roles [56]. 

Long-standing clinical trial results provide compelling proof-of-concept that vaccination can modulate the risk of CMV acquisition and possibly also the risk of CMV disease. The CMV strain Towne was generated by extensive serial virus passaging in fibroblasts and has been evaluated as a live-attenuated vaccine since the 1970s [57]. In a placebo-controlled trial, Towne was not able to protect seronegative mothers from acquiring CMV infection via exposure to their children attending daycare [58]. However, in a controlled challenge study in humans using an unattenuated CMV strain (Toledo) given subcutaneously (up to 1000 plaque-forming units), vaccination with Toledo demonstrated some degree of protection against infection and symptomatic illness (although to a lesser extent than protection conferred by natural immunity) [59]. In renal-transplant patients, Towne vaccination again failed to protect against CMV infection but provided significant protection against serious CMV disease [60]. 

Another notable landmark in CMV vaccinology is the subunit gB vaccine administered with MF59, an oil-in-water adjuvant. gB/MF59 has been developed by Sanofi (Chiron in the early stages) since the 1990s. Vaccination with gB/MF59 demonstrated protection against primary infection (ascertained by seroconversion to non-gB CMV antigens) of 50% (95% CI: 7 to 73%) in seronegative women [61] and 43% (95% CI: −36 to −76%; *p* = 0.20) in seronegative adolescent girls [62]. Furthermore. gB/MF59 provided significant protection against CMV DNAemia and a reduction in the number of days on antiviral therapy in solid organ transplant patients, particularly for seronegative recipients of organs from seropositive donors [63]. gB/MF59 induced both antibody and T-cell responses against gB; these responses were found to be boosted in seropositive immunocompetent individuals following vaccination (a finding with possible implications for the potential of vaccination to prevent congenital CMV infection in seropositive populations) [64]. Assessment of functional antibodies have historically focused on neutralizing antibodies. However, recent analyses of archived samples from vaccinees who participated in these gB/MF59 trials suggest that non-neutralizing antibodies may play a role in conferring protection [51,52]. 

Numerous companies are building on the promising leads generated by the Towne live-attenuated vaccine and the gB/MF59 subunit vaccine and are actively pursuing the development of CMV vaccines using various approaches. The tegument protein pp65 and the immediate early gene product IE1 are also considered as potential vaccine antigens because they are potent inducers of T-cell responses [65]. Strategic design intended to maximize or optimize immunogenicity is a necessary early step in vaccine development; however, establishing clinical evidence demonstrating whether that design effort translates to meaningful clinical benefit is not trivial and requires considerable time and resources. Regulatory issues that may be associated with CMV vaccine development, including possible clinical-study endpoints, have been recently reviewed [66].

Merck is developing a replication-defective, whole-virus vaccine known as V160 based on the laboratory strain AD169 with restored expression of PC [67]. Laboratory strains such as AD169 and Towne that have undergone extensive passaging in fibroblasts are invariably defective in PC expression due to poorly understood selective pressures [68]; the coding capacity for PC was restored to improve the immunogenicity of V160 (i.e., to be capable of inducing antibodies to PC). Further genetic engineering to make the stability of two essential viral gene products (IE1 and UL51; both expressed as fusions to the destabilizing domain of FK506-binding protein 12) dependent on the presence of an exogenous chemical compound (Shield-1) renders the vaccine virus to be capable of efficient replication in cell culture but incapable of replication in vivo [67]. The vaccination of rhesus macaques with V160 induced neutralizing antibodies to PC epitopes at levels comparable with those of CMV hyperimmune globulin, and the serum from vaccinated animals neutralized a panel of CMV clinical isolates as assessed in epithelial cells, thereby validating the successful reconstitution of PC as a key vaccine antigen in V160 [69]. Phase 1 evaluation of V160, formulated with or without aluminum phosphate adjuvant, demonstrated that the vaccine was well tolerated with no detectable shedding of the vaccine virus [70]. In addition, the levels of neutralizing antibodies and T-cell responses induced in vaccinated seronegative individuals were comparable with those following natural infection with CMV. The antibodies induced in humans were of high avidity (against gB, PC, and purified whole virus) with a neutralizing activity against diverse clinical CMV isolates demonstrated in a variety of cell types (epithelial, endothelial, cytotrophoblast, and neuronal cells). The induction of memory B cells at high frequency was also observed, suggesting durable humoral immunity [71]. In terms of cellular immune responses, polyfunctional CD4+ and CD8+ T cells to immunodominant antigens (IE1 and pp65) with a predominant effector phenotype were induced by V160 vaccination [72]. V160 is currently being evaluated in a Phase 2 study of seronegative women (NCT03486834).

Both Sanofi and GlaxoSmithKline (GSK) are pursuing adjuvanted subunit vaccines against CMV. The earlier efficacy findings with Sanofi’s gB/MF59 vaccine in seronegative women and transplant recipients were briefly discussed above. The Phase 1 immunogenicity results of GSK’s gB subunit vaccine adjuvanted with AS01E (liposomal formulation of monophosphoryl lipid A and the saponin QS21) were evaluated in 2011 with the results posted in ClinicalTrials.gov in 2017 [73]; anti-gB and neutralizing antibodies, gB-specific T cells (intracellular cytokine staining), and gB-specific memory B cells (ELISPOT) were measured. Both Sanofi and GSK are currently evaluating the addition of PC as a vaccine antigen, with the anticipated benefit being the induction of potent neutralizing antibodies that can inhibit the infection of epithelial/endothelial cells relative to a vaccine based on gB alone [74].

An approach based on virus-like particles (VLPs) may offer theoretical advantages in terms of how the vaccine antigen is presented to the immune system. An example is the enveloped VLP vaccine VBI-1501 developed by VBI Vaccines. VBI-1501 is generated by co-expressing the gB extracellular domain fused with the transmembrane and cytosolic domains of vesicular stomatitis virus G protein (termed gB-G) and the murine leukemia virus matrix protein Gag; the enveloped VLPs released into the culture medium from transfected cells are purified to generate the drug substance for VBI-1501 [75]. In cell culture, the expression of gB-G leads to cell-syncytia formation; the apparent fusogenicity of gB-G suggests that it may assume conformations resembling relevant structures of native gB [75]. A Phase 1 study of VBI-1501 administered with or without alum was undertaken in 2016 (NCT02826798) and immunogenicity results were posted in 2020 [76]. As expected, the neutralizing antibodies measured using fibroblasts were observed to be induced by VBI-1501, consistent with the indispensable role of gB in CMV entry for all cell types including fibroblasts. Notably, neutralizing antibodies measured using epithelial cells were also induced in some vaccinated individuals; this finding corroborates data from preclinical studies and contrasts with the low neutralizing activity in epithelial cells generally observed following vaccination with soluble gB [75]. The apparent expansion in the breadth of neutralizing activity induced by VBI-1501 may reflect the advantages associated with the structural features of membrane-anchored gB-G presented to the immune system in the context of enveloped VLPs.

There are examples of vaccine approaches exploiting viral vectors. Triplex, developed by City of Hope, is an attenuated modified vaccinia Ankara (MVA) poxvirus engineered to express three immunodominant CMV antigens (pp65, IE1-exon4, and IE2-exon5). The advantages of MVA include its demonstrated safety in various populations, notably hematopoietic stem-cell transplant (HSCT) recipients [77], the capacity of its genome to accommodate large inserts and its ability to induce robust immune responses. A first-in-human study of Triplex in healthy individuals was reported [78]. CMV-specific T cells were induced in both seropositive and seronegative healthy individuals regardless of prior exposure to smallpox vaccination. A randomized, placebo-controlled Phase 2 study of Triplex in seropositive HSCT recipients at high risk for CMV reactivation was recently published [79]. The vaccine, administered intramuscularly at days 28 and 56 after HSCT, was well tolerated with no vaccine-related serious adverse events. During the 100 days post-HSCT, reactivation of CMV was reduced in the vaccinated group (hazard ratio of 0.46; 95% CI of 0.16 to 1.4; *p* = 0.075). Levels of pp65-specific CD4+ and CD8+ T cells were higher in subjects receiving Triplex versus placebo. A Phase 2 study of Triplex evaluating whether vaccination of HSCT donors prior to donation can impact CMV viremia in recipients of donor stem-cell transplants is currently ongoing (NCT03560752). Another example using a viral-vector approach is HB-101, a bivalent vaccine based on replication-defective lymphocytic choriomeningitis virus vectors (LCMV) encoding gB or pp65, developed by Hookipa Pharma. LCMV may be an attractive vector for vaccine development because of its tropism for dendritic cells and its robust induction of CD8+ T cell responses [80]. The HB-101 vaccine viruses were engineered such that the native LCMV glycoprotein (GP) gene was replaced with genes encoding gB or pp65; the recombinant viruses are rescued (by transfection) and amplified using production cells expressing GP [81]. In the guinea pig model of congenital CMV infection, the gB and pp65 vectors exhibited additive benefits in terms of protection against maternal viremia and reduction in pup mortality compared with either vector alone [81]. The results from a Phase 1 study in healthy seronegative subjects were recently reported [82]. HB-101 was well-tolerated and immunogenic. Neutralizing antibodies (measured in fibroblasts) as well as polyfunctional gB- and pp65-specific CD8+ T cells (positive for IFN-γ and TNF-α as well as IL-2 and/or CD107a) were induced by vaccination. One out of 42 vaccinated subjects mounted a detectable neutralizing antibody response against LCMV, suggesting that anti-vector immunity may not pose a large obstacle for repeat boosting with HB-101. A Phase 2 study of HB-101 in kidney transplant patients is ongoing (NCT03629080). 

mRNA vaccines have recently generated considerable interest. The mRNA vaccines developed by Moderna and Pfizer-BioNTech against COVID-19 have unequivocally established the feasibility of this platform [83,84]. For mRNA vaccines, mRNA encoding the vaccine antigen (sequence/codon-optimized) is synthesized by large-scale in vitro transcription (often incorporating modified nucleosides to decrease innate immune activation and to increase translation) and subsequently purified; for many mRNA vaccines in advanced development, the resulting mRNA drug substance is encapsulated by lipid nanoparticles to formulate the vaccine drug product [85]. The lipid nanoparticles serve to protect the mRNA and to facilitate in vivo uptake by appropriate cells in which the mRNA payload is translated and presented to the immune system. Moderna is actively developing an mRNA vaccine against CMV. Preclinical data in mice and non-human primates have been published [86]. The delivery of multiple mRNAs encoding gB, PC, and pp65 resulted in durable humoral and cellular responses in animals. Neutralizing antibodies (measured in both epithelial cells and fibroblasts) were observed in vaccinated animals with titers comparable with or exceeding those associated with a lot of Cytogam used as a benchmark. Although the simultaneous administration of mRNAs encoding gB, PC, and pp65 appeared to result in epitope competition not favorable to pp65, sequential administration (priming with pp65 followed by boosting with gB + PC + pp65) in mice restored robust pp65 T-cell responses [86]. Moderna has focused on a vaccine candidate known as mRNA-1647, based on six mRNAs encoding gB and PC, for further clinical evaluation. Phase 1 (NCT03382405) and Phase 2 (NCT04232280) studies in healthy adults have been undertaken. Promising interim immunogenicity results have been announced by Moderna but are not yet published. Besides Moderna, other early CMV mRNA vaccine development efforts have been described. A generally promising variation is the use of self-amplifying mRNA (SAM) replicons based on engineering the genome of positive-sense, single-stranded RNA viruses (notably alphaviruses) such that the coding region for viral structural genes are replaced with genes coding for vaccine antigens [87]. SAMs may be associated with potential benefits such as increased potency and more persistent in vivo expression. It is also possible to encode multiple gene products within one SAM construct; this property may be attractive in the context of CMV vaccine development, which likely requires targeting multiple vaccine antigens. The feasibility of encoding the five genes necessary for PC in a single SAM replicon (using alphavirus subgenomic promoters and internal ribosome entry sites) has been reported; the proper translation of PC and induction of potent neutralizing antibodies in mice have been demonstrated [88].

### 3.2. Polyclonal Antibody Therapy

Hyperimmune globulins (HIG) are enriched for antibodies against a specific virus or bacterium and are purified from the plasma of convalescent or vaccinated donors. This strategy has been widely used since the 1940s when the first human derived antiviral HIG products were licensed for the prevention of measles and hepatitis A. Additional hyperimmune IG products were licensed over time for the prevention of rabies, hepatitis B, varicella, vaccinia and CMV. Cytomegalovirus immune globulin (Human)(Cytogam) was licensed in the US in 1990 [54]. A similar product, Cytotect CP Biotest, is licensed in Europe [89].

Hyperimmune plasma for Cytogam and Cytotect (CMVIgG) is selected from normal human plasma from qualified blood or plasma donors [54,90]. Although an estimated 49–74% of adults (aged 20–59) in the US are seropositive for CMV [91], antibody levels are variable. Historically, about one in fifteen donations had CMV titers considered potent enough to pool for manufacturing of a hyperimmune product [92]. Cytogam is manufactured using a modified Cohn (method 6) [93] and Oncley (method 9) [94] ethanol precipitation process. Cytotect is also purified using ethanol fractionation. Both products are formulated as a 5% IgG; Cytogam is stabilized with 5% sucrose [54] and Cytotect with glycine [90]. As for most antiviral HIG products, the potency of these products is measured using a neutralization assay. There have been episodic publications suggesting that normal IG products could substitute for hyperimmune CMVIG products [95,96,97] because CMV seropositivity is common. Direct comparisons of CMVIgG with non-hyperimmune products have shown that the former have consistently higher levels of anti-CMV antibodies and neutralizing activity compared with intravenous IgG preparations [90,98,99].

Viral neutralization is clearly of major importance in humoral responses to CMV, but there is evidence for non-neutralizing effector functions as well. These include complement-mediated cytotoxicity and antibody-dependent cellular cytotoxicity [100,101,102]. It is not clear whether, or to what extent, these mechanisms may play a role in the efficacy of CMVIgG.

CMVIgGs are indicated for prophylaxis of CMV disease associated with transplantation of kidney, lung, liver, pancreas and heart. For all solid organ transplants, except kidneys, those involving CMV-seropositive donors and CMV-seronegative recipients, prophylactic Cytogam should be considered in combination with ganciclovir [54]. Cytotect is licensed in UK and other European countries for “Cytomegalovirus (CMV) infection prevention in patients subjected to immunosuppressive therapy, particularly in transplant recipients [89]”. In the 1980s and early 1990s, CMVIgG was studied as a monotherapy. The advent of antiviral treatments heralded the use of combination therapy with CMVIgG and ganciclovir or similar antivirals [103]. Recent evidence-based consensus guidelines for the management of CMV in solid organ transplantation emphasize pre-emptive antiviral use for the prevention of severe CMV in high risk recipients, but also note that “some experts add CMV-Ig” to antiviral regimens in the case of liver, heart, and small bowel transplantation [104]. 

Cytomegalovirus fetal infections are a result of vertical transmission and occur in up to 40% of births if the mother has had a primary CMV infection during pregnancy [105]. The frequency of transmission is much lower (less than 4%) in the case of maternal reactivation of CMV during pregnancy. Severe fetal outcomes can occur if infection is transmitted, especially during the first trimester. These can include microcephaly, sensorineural hearing loss, developmental delays, neurocognitive deficits, low birth weight, and vision loss. Treatment with CMVIgG has been studied for the prevention of fetal infection. Initial non-randomized studies suggested that this approach could be effective. A Phase 2 randomized controlled blinded trial evaluated CMVIgG in primary CMV infection during pregnancy. Congenital infection was present in 30% of the treatment group and 44% of the control group, a non-significant difference [106]. Critics have pointed out that the median time interval of 5 weeks between diagnosis of primary CMV infection and CMVIgG treatment allowed sufficient time for viral transmission [107]. Hyperimmune globulins in general are most effective when given before or shortly after viral infection–when the viral load is low and before endogenous adaptive immune responses have had time to develop. A second caveat is related to the low dose and monthly dosing intervals, which may not have been sufficient in the pregnancy setting where the half-life of IG can be shorter [107,108,109]. At this time, the use of small molecule antivirals such as valaciclovir or CMVIgG to prevent congenital CMV remains controversial [109,110], and it is hoped that additional studies will be performed. 

### 3.3. Monoclonal Antibody Therapy

The use of HCMV-specific antibodies for the prevention of HCMV infection and disease after HSCT or solid-organ transplant (SOT) has been studied for some time. Despite these efforts, there are no HCMV mAbs that have been FDA-approved. There are several mAbs that have been evaluated in clinical trials and are briefly summarized below.

MSL-109 is a human monoclonal antibody directed against HCMV surface glycoprotein gH. MSL-109 neutralizes both laboratory and clinical strains of HCMV in cell culture [111]. MSL-109 was tested as an adjuvant treatment for AIDS patients with HCMV-induced retinitis, but development was halted during Phase 2/3 clinical trials when data showed that MSL-109 lacked sufficient efficacy [112]. While a transient reduction in HCMV DNAemia in newly diagnosed patients with HCMV retinitis was observed, they relapsed at 6 months.

RG7667 consists of a combination of two monoclonal antibodies that bind to neutralizing epitopes on the HCMV complexes gH/gL and gH/gL/UL128/UL130/UL131 [113]. RG7667 neutralizes HCMV infection of all the cell types tested. RG7667 has been evaluated in a Phase 2 randomized, double-blind, placebo-controlled trial in kidney-transplant recipients [114] (NCT01753167). The proportion of patients exhibiting the primary endpoint of HCMV DNAemia within 12 weeks posttransplant was lower in the RG7667 group (27 of 59 (45.8%)) than in the placebo group (35 of 57 (61.4%)). However, the stratum-adjusted difference (15.3%) was not significant at the unadjusted 5% level of significance (95% confidence interval [CI], −2.8% to 32.2%; *p* = 0.100). A significantly lower proportion of patients in the RG7667 group developed CMV disease within 24 weeks post-transplant than in the placebo group (2 of 59 (3.4%) versus 9 of 57 (15.8%), respectively; *p* = 0.030).

CSJ148 consists of two anti-HCMV human monoclonal antibodies (LJP538 and LJP539) [115]. Each antibody binds to and inhibits the function of essential viral glycoproteins; LJP538 binds to glycoprotein B (gB), and LJP539 binds to the pentameric complex (consisting of glycoproteins gH, gL, UL128, UL130, and UL131). CSJ148 neutralizes HCMV infection of all the cell types tested by blocking both initial infection and the subsequent cell-to-cell spread of virus [115]. CSJ148 was evaluated in a Phase 2, randomized, placebo-controlled trial for prophylaxis of HCMV in patients undergoing allogeneic hematopoietic stem cell transplantation [116] (NCT02268526). The primary efficacy endpoint was not met as the estimated probability that CSJ148 decreases the need for preemptive therapy compared with placebo was 69%, with a risk ratio of 0.89 and a 90% credible interval of 0.61 to 1.31. CSJ148-treated patients showed trends toward decreased viral load, shorter median duration of preemptive therapy, and fewer courses of preemptive therapy.

## 4. Discussion and Conclusions

Although HCMV infection remains a public-health burden worldwide, successful vaccines have been elusive and effective therapies scant. CMVIgG polyclonal antibody products have been approved for the prevention of CMV in SOT setting, but clinical trials for their use during pregnancy have been inconclusive. Similarly, lackluster results from clinical trials have precluded the approval of monoclonal antibodies for the treatment of CMV infection and disease. The lack of a directly translatable animal model for HCMV represents a significant barrier in the development of CMV immunotherapies. Together with existing models, the recent development of HCMV infection in HIS humanized mice should greatly assist in the discovery and successful translation of treatment and prophylactic modalities for CMV disease. The recent successes of the clinical trials in Ebola virus disease [117] and SARS-CoV-2 infections should encourage a renewed interest in this class of therapeutics, which may hold great potential in the prevention and treatment of CMV. There is numerous CMV vaccine development efforts in progress. The examples we presented are by no means exhaustive; rather, they serve to highlight the variety of approaches and platforms in play, each with features that may be beneficial for the intended indication being pursued. Clinical data that corroborate and extend early proof-of-concept results achieved with the Towne live-attenuated vaccine and the gB/MF59 subunit vaccine are eagerly awaited.

## Figures and Tables

**Table 1:** Vaccines and Antibody Therapies for CMV *.

	Licensed for Marketing (USA)	Registered in clinicaltrials.gov (accessed on 15 June 2021)	
		Total Trials (Completed)	Unique Molecular Entities
**Vaccines**	None	21 (9)	14
**Monoclonal antibodies**	None	1 (0)	1
**Polyclonal antibodies**	Cytogam	2 (1)	1

* Clinicaltrials.gov webpage was searched for interventional studies that included keyword “CMV”. The list was manually curated to select studies for CMV indications with biologics but excluded T cells, CTL or adoptive transfer therapies. Studies that were (or projected to be) completed after 2011 were counted for this table.
